# UBTD1 Drives Ovarian Cancer Progression via Mutation‐Associated Alterations, Stromal Microenvironment Remodeling, and TNF/AP‐1 Signaling

**DOI:** 10.1155/humu/9479429

**Published:** 2026-07-22

**Authors:** Aixin Liu, Yanxia Chen, Xian Zhao, Junying Zhou, Guanying Feng, Huijuan Zhang, Baiyang Liu, Yongbin Chen, Cuiping Yang

**Affiliations:** ^1^ The International Peace Maternity and Child Health Hospital, School of Medicine, Shanghai Jiao Tong University, Shanghai, China, sjtu.edu.cn; ^2^ Shanghai Key Laboratory of Embryo Original Diseases, Shanghai, China, sjtu.edu.cn; ^3^ The First Affiliated Hospital of Zhengzhou University, Zhengzhou, China, zzu.edu.cn; ^4^ State Key Laboratory of Genetic Evolution and Animal Models, The Key Laboratory of Animal Models and Human Disease Mechanisms of Yunnan Province, Kunming Institute of Zoology, Chinese Academy of Sciences, Kunming, China, cas.cn

**Keywords:** ovarian cancer, TNF/AP-1 signaling, tumor microenvironment, tumor mutation burden, UBTD1

## Abstract

**Background:**

The ubiquitin domain‐containing protein 1 (UBTD1) is involved in protein homeostasis and cell cycle regulation, and emerging evidence suggests its role in tumor biology. However, its function in ovarian cancer (OC) remains unclear. OC is highly lethal due to late diagnosis, metastasis, and platinum resistance. This study is aimed at investigating the role of UBTD1 in OC by integrating single‐cell transcriptomics, mutation and HRD‐related analyses, tumor microenvironment evaluation, and experimental validation.

**Methods:**

We examined UBTD1 expression, prognosis, somatic mutation features, and immune microenvironment characteristics utilizing public databases, such as the Cancer Genome Atlas (TCGA) and Gene Expression Omnibus (GEO). Single‐cell RNA‐seq data were analyzed using Seurat, hdWGCNA, and CopyKAT to characterize UBTD1‐associated malignant‐cell states and inferred CNV burden. Based on the median UBTD1 expression, TCGA‐OV samples were divided into high and low expression groups to compare mutation spectra, DNA repair pathway activity, stromal scores, and immune cell infiltration. Functional assays were performed in A2780 and SK‐OV‐3 cell lines following lentiviral shRNAs‐mediated UBTD1 knockdown. RNA sequencing and rescue experiments were used to explore downstream pathways.

**Results:**

Integrated single‐cell and TCGA‐OV analyses revealed that UBTD1‐low tumors were enriched for genomic instability–related features, including increased inferred CNV burden and higher tumor mutation burden. By comparison, UBTD1‐high tumors showed increased stromal scores and modest enrichment of selected immune‐cell populations, including macrophages and neutrophils. Experimentally, UBTD1 was upregulated in OC and associated with poor prognosis. UBTD1 knockdown inhibited malignant phenotypes, enhanced cisplatin sensitivity, promoted apoptosis, and suppressed TNF/AP‐1/FOS–related signaling. FOS overexpression partially reversed the effects of UBTD1 silencing.

**Conclusions:**

UBTD1 expression defines distinct mutation and microenvironmental states in OC and functionally promotes malignant phenotypes, potentially through TNF/AP‐1/FOS–related signaling.

## 1. Introduction

Globally, OC represents a substantial cause of cancer mortality in women [1]. The poor outcome of OC is largely related to its tendency to be identified only after the disease has already progressed to advanced stages, with nearly 70% of cases diagnosed at International Federation of Gynecology and Obstetrics (FIGO) Stages III–IV [2]. This pattern is closely associated with the absence of dependable early screening approaches, together with the deep pelvic location of the ovaries. As a result, the overall 5‐year survival rate remains only about 50% [3]. Current standard management usually combines cytoreductive surgery with platinum‐based chemotherapy, often leading to an initial clinical response [4]. Nevertheless, recurrence eventually occurs in most patients, and the relapsed disease is frequently no longer sensitive to platinum‐based therapy [5–9]. This clinical challenge makes it important to further dissect the molecular basis of ovarian cancer progression and platinum resistance.

The UBTD protein family, including UBTD1 and UBTD2, functions as regulatory components within the ubiquitin‐proteasome system (UPS) [10–16]. UBTD1 modulates protein stability by interacting with E2 ubiquitin‐conjugating enzymes [17]. Intriguingly, UBTD1 exhibits context‐dependent roles in cancer, exerting oncogenic activity in colorectal cancer but acting as a tumor suppressor in prostate and lung cancers [11, 18]. Its function in ovarian cancer remains elusive.

In this work, we aim to comprehensively characterize the role of UBTD1 in ovarian cancer from the perspectives of mutation, tumor microenvironment remodeling, and functional tumor progression. We first integrated single‐cell RNA sequencing data, TCGA‐OV transcriptomic and somatic mutation profiles, and tumor microenvironment analyses to determine whether UBTD1 expression was associated with malignant‐cell states, mutation burden, and stromal‐immune features. We then assessed UBTD1 expression patterns, clinicopathological associations, and prognostic relevance using public datasets, followed by lentiviral knockdown assays and transcriptomic/FOS rescue analyses to evaluate its functional and mechanistic roles in ovarian cancer.

Our findings suggest that UBTD1 is associated with both genomic instability and tumor microenvironment remodeling in ovarian cancer. UBTD1‐low tumors were characterized by increased inferred CNV burden, higher tumor mutation burden, and elevated HRD scores, whereas UBTD1‐high tumors showed increased stromal scores and modest enrichment of macrophages and neutrophils. Functional experiments further showed that UBTD1 promotes ovarian cancer proliferation, migration, invasion, and cisplatin resistance, potentially through TNF/AP‐1/FOS–related signaling. Collectively, these results support a multifaceted role of UBTD1 in ovarian cancer progression, linking genomic instability, stromal‐immune remodeling, and malignant tumor phenotypes.

## 2. Materials and Methods

### 2.1. Cell Lines and Culture Conditions

IOSE‐80, an immortalized ovarian epithelial cell line, was purchased from Shanghai Longli Biotechnology Co. Ltd. HEK‐293T, SK‐OV‐3, OVCAR‐3, and Caov‐3 cells were obtained from the Cell Bank of the Chinese Academy of Sciences, whereas A2780 cells were purchased from Shanghai Sheng′er Biotechnology Co. Ltd. STR profiling was used to confirm the identity of all cell lines.

For routine culture, SK‐OV‐3, OVCAR‐3, Caov‐3, and A2780 cells were grown in DMEM/F12 (Cat. C11330500CP, Gibco) containing 10% FBS (Cat. A5256701, Gibco) together with 1% penicillin–streptomycin (Cat. G4003, Gibco). HEK‐293T and IOSE‐80 cells were instead propagated in high‐glucose DMEM (Cat. C11995500CP, Gibco) supplemented with the same additives. All cultures were kept in a humidified 5% CO_2_ atmosphere at 37°C.

### 2.2. Construction, Transfection, and Lentiviral Infection

Independent shRNAs targeting UBTD1 were cloned into pLKO.1 vector, with all recombinant constructs verified by DNA sequencing. For lentiviral generation, HEK‐293T cells were introduced with the control shRNA construct or the UBTD1‐knockdown construct, together with psPAX2 and pMD2.G, by a calcium phosphate–based transfection approach [19, 20]. Lentiviral supernatants were collected at 48 and 72 h posttransfection, centrifuged to remove cell debris, and filtered through a 0.45‐*μ*m filter. For infection, target ovarian cancer cells were incubated with viral supernatants supplemented with polybrene (8 *μ*g/mL) to enhance infection efficiency. After 8–12 h, the medium was replaced with fresh complete medium. Stable UBTD1‐knockdown cell lines were selected using puromycin (Cat. A1113803, Gibco) at an appropriate concentration for 3–5 days until noninfected control cells were completely eliminated.

The FOS overexpression plasmid (FOS‐pcDNA3.1–3 × flag‐C, Shanghai Longli Biotechnology) and the corresponding empty vector (pcDNA3.1) were individually transfected into specified cell lines using Lipofectamine 3000 (Cat. L3000015, Invitrogen), followed by functional assays.

### 2.3. Immunoblotting and qRT‐PCR Analysis

Protein abundance and mRNA expression were determined by immunoblotting and qRT‐PCR, respectively. These techniques provided insights into the molecular mechanisms under investigation.

Total cellular proteins were extracted using RIPA lysis buffer (Beyotime, Cat. P0013B) for immunoblotting analysis containing a protease inhibitor cocktail (Roche, Cat. 11836170001). Proteins were extracted, separated using SDS‐PAGE (sodium dodecyl sulfate–polyacrylamide gel electrophoresis), transferred onto PVDF (polyvinylidene fluoride) membranes, and immunoblotted with indicated antibodies following standard protocols [21].

Total RNA was isolated using Vezol reagent (Cat. R411‐01, Vazyme). Reverse transcription was subsequently carried out with the Vazyme kit (Cat. RT101‐01, Vazyme) to generate cDNA. Quantitative PCR was then run on a QuantStudio 5 system with ChamQ Blue Universal SYBR qPCR Master Mix (Cat. Q312‐02, Vazyme). Gene expression levels were standardized using *β*‐actin as a reference. Antibodies, primers, and oligonucleotides are listed in Tables S1 and S2.

### 2.4. Growth Curve, Colony Formation, and BrdU Incorporation

In the growth curve assay, 5 × 10^4^ indicated cells were plated into each well of six‐well plates (Cat. 3516, Corning), and cell numbers were measured every day with a Countstar Mira BF automated counter.

For colony formation analysis, 1000 indicated cells were seeded per well in six‐well plates and cultured under standard conditions for 10–14 days until macroscopically visible colonies were formed [22]. The culture medium was replaced every 3 days. Colonies were preserved using 4% paraformaldehyde (4% PFA; Cat. BL1279A, Biosharp), and stained using 0.1% crystal violet (Cat. BL802B, Biosharp). Manually count colonies composed of 50 or more cells under a microscope for quantitative analysis.

For 5‐bromo‐2 ^′^‐deoxyuridine (BrdU; Cat. B23151, Invitrogen) incorporation assay, cells were subjected to a 20‐min pulse with 10 *μ*M BrdU, followed by fixation and immunostaining with an anti‐BrdU antibody (Cat. AB6326, Abcam), and counterstaining with 4 ^′^, 6‐diamidino‐2‐phenylindole (DAPI; Servicebio, G1012 100ML). BrdU‐positive nuclei were imaged with a fluorescence microscope (BX43FC, OLYMPUS), and positive cells were determined in five randomly selected microscopic fields for each sample.

### 2.5. Wound Healing Assay

After the cells reached confluence in six‐well plates, a straight scratch was made across the monolayer with a sterile pipette tip to evaluate migratory capacity. After washing with PBS (phosphate‐buffered saline), wound closure was monitored at regular intervals by inverted microscopy (DMi8, Leica). For quantification, images were analyzed using ImageJ software. The wound area at each time point was manually outlined to generate a closed region, and the corresponding area was measured. The percentage of wound closure was calculated using the following formula: Wound closure (*%*) = (initial wound area − wound area at indicated time point)/initial wound area × 100*%*.

### 2.6. Transwell Migration and Invasion assay

For the migration experiment, each upper insert of the 24‐well Transwell insert (Cat. 3422, Corning) received 4 × 10^4^ cells in 200 *μ*L of serum‐free medium. Meanwhile, 600 *μ*L complete medium supplemented with 10% FBS was placed in the lower compartment to provide a chemotactic stimulus. Cell migration was quantified by fixing cells on the lower membrane surface with 4% PFA, staining with 0.1% crystal violet after 24 h, and counting them in five random fields per membrane.

In the invasion assay, matrigel‐coated (Cat. 356234, Corning) transwell inserts were utilized and solidified into a continuous gel membrane at 37°C. Subsequently, 7 × 10^4^ cells in 200 *μ*L of serum‐free medium were placed in the upper chamber, whereas 600 *μ*L of complete medium supplemented with 10% FBS was added to the lower chamber. After a 48‐h invasion period, the cells that had traversed to the underside of the membrane were then subjected to fixation and staining. Quantification was then performed as previously described for the cell migration assay.

### 2.7. Flow Cytometry Analysis

To determine cell cycle distribution, harvested cells were washed with PBS and then fixed in 75% ethanol at 4°C overnight. After the fixation step, cells were stained using propidium iodide (PI; Cat. C1052, Beyotime) containing RNase (Cat. C1052, Beyotime) and incubated at 37°C for 30 min in darkness. Cell cycle distribution was determined on a CytoFLEX LX (Beckman Coulter). For apoptosis analysis, cells were exposed to cisplatin (Cat. P4394, Sigma) for 72 h, then carefully collected and stained using an Annexin V‐FITC (fluorescein isothiocyanate)/PI apoptosis detection kit (Cat. 556547, BD). Samples were analyzed using the FACSAria SORP flow cytometer (BD, Bioscience) with quadrants established using unstained and single‐stained controls. The overall apoptosis rate was determined by adding the early and late apoptotic cell counts.

### 2.8. Single‐Cell RNA‐Seq Analysis and UBTD1 Virtual Knockdown Analysis

Single‐cell RNA‐seq data from GSE184880 were processed using Seurat. After quality control, normalization, PCA, Harmony‐based batch correction, clustering, and cell‐type annotation, epithelial cells were analyzed using CopyKAT to infer genome‐wide copy‐number alterations, and aneuploid cells were defined as malignant cells. UBTD1‐positive malignant cells were defined as malignant cells with detectable UBTD1 expression. hdWGCNA was performed in malignant cells, and the UBTD1‐associated module was used for GO and KEGG enrichment analyses.

For expression‐based in silico UBTD1‐low/virtual knockdown–like analysis, UBTD1‐positive malignant cells were divided into UBTD1‐high/active–like and UBTD1‐low/virtual knockdown–like cells according to the upper and lower quartiles of UBTD1 expression, respectively. Cells in the middle 50% were excluded. Genomic instability–related transcriptional programs were quantified using UCell module scoring. CopyKAT‐inferred CNV burden was calculated from inferred copy‐number profiles and compared between UBTD1‐high and UBTD1‐low cells using the Wilcoxon rank‐sum test.

### 2.9. TCGA‐OV Somatic Mutation, HRD, and Pathway Analyses

For TCGA‐OV analyses, samples were stratified into UBTD1‐high and UBTD1‐low groups according to median UBTD1 expression. Somatic mutation data were analyzed using maftools to compare tumor mutation burden, SNV classes, and top mutated genes. ssGSEA was used to calculate homologous recombination, DNA repair, macrophage, and neutrophil enrichment scores. Stromal scores were calculated using ESTIMATE. HRD scores and HRD‐high status were integrated with TCGA‐OV expression data and compared between UBTD1 groups.

### 2.10. Mutational Signature and Tumor Microenvironment Analyses

For SBS mutational signature analysis, TCGA‐OV somatic SNVs were used to construct a 96‐channel trinucleotide mutation matrix and estimate SBS signature contributions. The HRD‐related SBS3 contribution was compared between UBTD1 groups and correlated with UBTD1 expression and HRD status. Group comparisons were performed using the Wilcoxon rank‐sum test or Fisher′s exact test, and correlations were assessed using Spearman′s correlation analysis. Multiple testing correction was performed using the Benjamini–Hochberg method where applicable.

Tumor microenvironment features were analyzed in TCGA‐OV samples. Stromal scores were calculated using the ESTIMATE algorithm. Macrophage and neutrophil infiltration scores were quantified using ssGSEA. Differences in stromal scores and immune‐cell infiltration scores between UBTD1‐high and UBTD1‐low groups were assessed using the Wilcoxon rank‐sum test. Spearman′s correlation analysis was used to evaluate associations between continuous UBTD1 expression and tumor microenvironment scores. Unless otherwise specified, multiple testing correction was performed using the Benjamini–Hochberg method, and adjusted *p* values < 0.05 were considered statistically significant.

### 2.11. Bioinformatic and Statistical Analysis

Gene expression and correlation analyses were conducted by GEPIA2 (http://gepia2.cancer-pku.cn/). Using the “Expression DIY” analysis tool, UBTD1 mRNA expression was compared between ovarian cancer samples and the nontumor tissues. Tumor samples were acquired from the TCGA ovarian cancer datasets, whereas normal ovarian tissue samples were obtained from the GTEx database. Pairwise correlations between UBTD1 and target genes were evaluated using Pearson correlation analysis.

The correlation between UBTD1 expression and key clinicopathological parameters (e.g., tumor stage and grade) was interrogated via the Hiplot Biomedical AI Platform, specifically utilizing its BEST database module. The diagnostic efficacy of UBTD1 was assessed using the receiver operating characteristic (ROC) Plotter platform (https://rocplot.org/), which generated a ROC curve and calculated the area under the curve (AUC).

Statistical analyses were conducted using both nonparametric and parametric methods. The Mann–Whitney *U* test and the Wilcoxon signed‐rank test were applied. A two‐tailed Student′s *T*‐test was used for comparing groups. Analyses were conducted using GraphPad Prism (Version 10), with statistical significance defined as *p* < 0.05. Significance levels were indicated as follows: ∗*p* < 0.05, ∗∗*p* < 0.01, ∗∗∗*p* < 0.001, ∗∗∗∗*p* < 0.0001, and “ns” for no significant difference.

## 3. Results

### 3.1. Single‐Cell Analysis Links Reduced UBTD1 Expression to Copy‐Number Alteration Burden and DNA Repair/Checkpoint Programs in Malignant Cells

**Figure 1:**
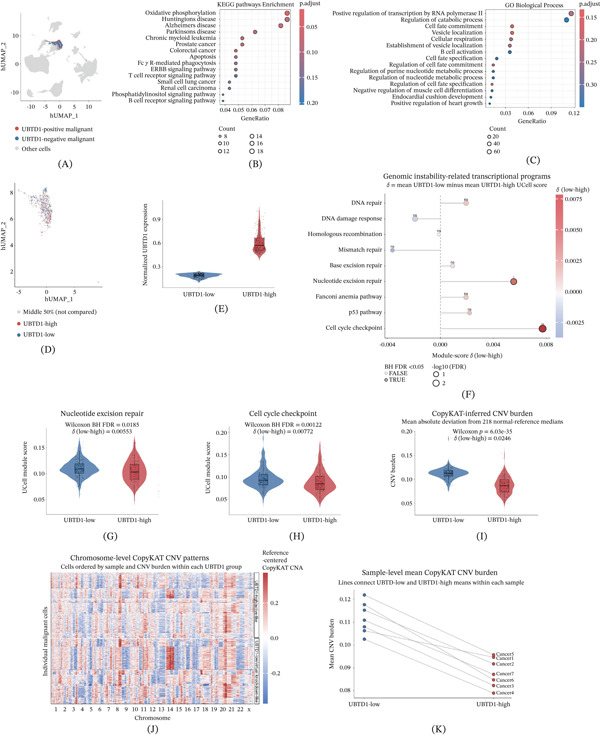
Single‐cell analysis links reduced UBTD1 expression to copy‐number alteration burden and DNA repair/checkpoint programs in malignant cells. (A) UMAP visualization of UBTD1‐positive and UBTD1‐negative malignant cells in the GSE184880 scRNA‐seq dataset. (B) KEGG enrichment analysis of genes from blue module identified via hdWGCNA analysis. (C) GO biological process enrichment analysis of genes from the blue hdWGCNA module in ovarian cancer malignant cells. (D) UMAP visualization of UBTD1‐high (*n* = 167) and UBTD1‐low (*n* = 167) malignant cells within UBTD1‐positive malignant cells. Cells in the middle 50% of UBTD1 expression were excluded from the high–low comparison. (E) Violin plot showing UBTD1 expression in UBTD1‐low (*n* = 167) and UBTD1‐high (*n* = 167) malignant cells. (F) Summary dot plot of mutation‐related DNA repair and checkpoint transcriptional programs in UBTD1‐low (*n* = 167) versus UBTD1‐high (*n* = 167) malignant cells. Dot color indicates the mean score difference, dot size indicates −log10(FDR), and black outlines indicate FDR < 0.05. (G–H) (G) Nucleotide excision repair and (H) cell cycle checkpoint module scores in UBTD1‐low (*n* = 167) and UBTD1‐high (*n* = 167) malignant cells. (I) CopyKAT‐inferred CNV burden in UBTD1‐low (*n* = 167) and UBTD1‐high (*n* = 167) malignant cells. (J) Chromosome‐level CopyKAT heat map showing reference‐centered copy‐number alteration patterns in UBTD1‐high (*n* = 167) and UBTD1‐low (*n* = 167) malignant cells. (K) Sample‐level mean CopyKAT‐inferred CNV burden in UBTD1‐low (*n* = 167) and UBTD1‐high (*n* = 167) malignant cells. Lines connect paired values from the same patient sample. (G–I) Statistical comparisons were performed using the Wilcoxon rank‐sum test.

To explore whether UBTD1 expression was associated with mutation‐related malignant‐cell states at the single‐cell level, we analyzed the GSE184880 ovarian cancer scRNA‐seq dataset. At the single‐cell level, scRNA‐seq data were reprocessed with Seurat and integrated using Harmony to reduce sample‐specific batch effects. Harmony‐integrated UMAP showed partial overlap between cancer‐derived and normal‐derived nonmalignant cells while preserving tumor‐associated differences (Figure S1A–B). Major cell populations were annotated based on canonical marker genes (Figure S1C). Epithelial/tumor–like cells were analyzed using CopyKAT for CNV inference. Cells with high CNV and aneuploid profiles were defined as malignant cells following standard CopyKAT criteria and visualized on the Harmony UMAP (Figure S1D–E). Among 2636 malignant cells, 670 were UBTD1‐positive and were used for downstream functional analysis (Figure 1A).

We performed hdWGCNA in CopyKAT‐confirmed malignant cells to identify UBTD1‐associated coexpression modules. Module eigengene analysis showed that the blue module had the strongest statistically significant positive correlation with UBTD1 expression after FDR correction (Figure S1F). A scale‐free network was constructed using an optimized soft‐thresholding power (Figure S1G). Genes in the blue module were therefore selected for KEGG and GO enrichment analyses, which revealed enrichment in cellular respiration, vesicle localization, transcriptional regulation, oxidative phosphorylation, and cancer‐related pathways (Figure 1B,C).

To further characterize the cellular features associated with reduced UBTD1 expression, UBTD1‐positive malignant cells were divided into UBTD1‐high (UBTD1 active‐like) and UBTD1‐low (UBTD1 virtual knockdown‐like) cells according to UBTD1 expression quartiles (Figure 1D,E). Compared with UBTD1‐high cells, UBTD1‐low cells showed selective activation of mutation‐related DNA repair and checkpoint transcriptional programs (Figure 1F). In particular, nucleotide excision repair and cell cycle checkpoint scores were significantly increased in UBTD1‐low cells (Figure 1G,H).

We next evaluated CopyKAT‐inferred copy‐number alteration burden as a single‐cell–level indicator of mutation‐associated genomic alterations. UBTD1‐low malignant cells exhibited markedly higher inferred CNV burden than UBTD1‐high cells (Figure 1I). Chromosome‐level CopyKAT profiles further showed broad copy‐number alteration patterns in malignant cells (Figure 1J), and sample‐level mean CNV burden analysis indicated that the increase in UBTD1‐low cells was observed across patient samples rather than being driven by a single sample (Figure 1K). Together, these findings suggest that the UBTD1‐low malignant‐cell state is associated with mutation‐related genomic alteration features, including enhanced DNA repair, checkpoint programs, and increased inferred copy‐number alteration burden.

### 3.2. UBTD1‐Low Tumors Show Increased Mutation Burden and HRD‐Related Genomic Features in TCGA‐OV

**Figure 2:**
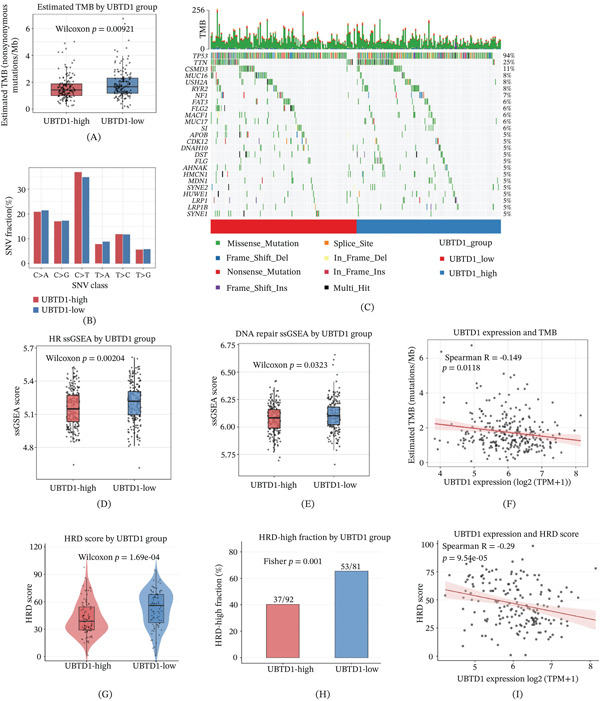
UBTD1‐low tumors show increased mutation burden and HRD‐related genomic features in TCGA‐OV. (A) Comparison of tumor mutation burden (TMB) between UBTD1‐high (*n* = 141) and UBTD1‐low (*n* = 143) groups. *p* value was calculated using the Wilcoxon rank‐sum test. (B) Distribution of six major single‐nucleotide variant (SNV) classes in UBTD1‐high (*n* = 141) versus UBTD1‐low (*n* = 143) groups. (C) Oncoplot displaying the top mutated genes in all samples, with UBTD1‐high (*n* = 141) and UBTD1‐low (*n* = 143) subgroups labeled accordingly. (D–E) ssGSEA scores of (D) homologous recombination and (E) DNA repair pathways in UBTD1‐high (*n* = 141) and UBTD1‐low (*n* = 143) tumors. (F) Correlation between UBTD1 expression and TMB. (G) HRD scores in UBTD1‐high (*n* = 92) and UBTD1‐low (*n* = 81) tumors. (H) Proportion of HRD‐high (*n* = 92) and HRD‐low (*n* = 81) tumors in the two UBTD1 expression groups. (I) Correlation between UBTD1 expression and HRD score. TMB, pathway scores, and HRD scores were compared using the Wilcoxon rank‐sum test. HRD‐high proportions were compared using Fisher′s exact test. Correlations were assessed using Spearman′s correlation analysis.

To investigate the association between UBTD1 expression and genomic alterations, TCGA‐OV samples were stratified into UBTD1‐high (*n* = 141) and UBTD1‐low (*n* = 143) subgroups according to the median expression level of UBTD1 (Figure S2A). Samples in the UBTD1‐low group exhibited a modestly higher tumor mutational burden (TMB) than those in the UBTD1‐high group (Figure 2A). Further analysis of somatic mutation patterns revealed that C > T transitions were the most prevalent SNV type in both groups (approximately 35% in the high group vs. 34% in the low group), followed by C > A and C > G substitutions, resulting in highly similar global SNV spectra between the two subgroups (Figures 2B and S2B). Consistently, the TCGA‐OV cohort showed a mutation landscape dominated by missense mutations and C > T substitutions, with TP53 as the most frequently mutated gene and substantial intersample heterogeneity in mutation burden (Figure S2C).

Oncoplot visualization of the top mutated genes (Figure 2C) further confirmed that the overall somatic mutation landscapes were largely comparable between UBTD1‐high and UBTD1‐low groups, with TP53 remaining the most frequently mutated gene in both subgroups (Figures 2C and S2D). Differential mutation analysis using Fisher′s exact test further revealed no markedly enriched genes between the two groups.

To further characterize genomic instability–related features associated with UBTD1 expression, ssGSEA was performed using Reactome pathway gene sets in the TCGA‐OV expression cohort. Compared with the UBTD1‐high group (*n* = 217), the UBTD1‐low group (*n* = 217) displayed significantly elevated enrichment scores for homologous recombination and DNA repair pathways (Figure 2D–E), suggesting enhanced DNA damage response and repair activity. In addition, UBTD1 expression was negatively correlated with TMB across TCGA‐OV samples (Figure 2F), further supporting a potential association between reduced UBTD1 expression and genomic instability–related characteristics.

We further evaluated whether UBTD1 expression was associated with HRD‐related genomic scar features. UBTD1‐low tumors had significantly higher HRD scores than UBTD1‐high tumors (Figure 2G). In line with this result, the proportion of HRD‐high cases was also higher in the UBTD1‐low group than in the UBTD1‐high group (Figure 2H). Continuous correlation analysis further showed that UBTD1 expression was negatively correlated with HRD score (Figure 2I). Together, these data suggest that low UBTD1 expression is associated with increased mutation burden, enhanced DNA repair/HR pathway activity, and HRD‐related genomic features in ovarian cancer.

### 3.3. UBTD1 Expression Is Associated With SBS3 Mutational Signature and Stromal‐Immune Remodeling in Ovarian Cancer

**Figure 3:**
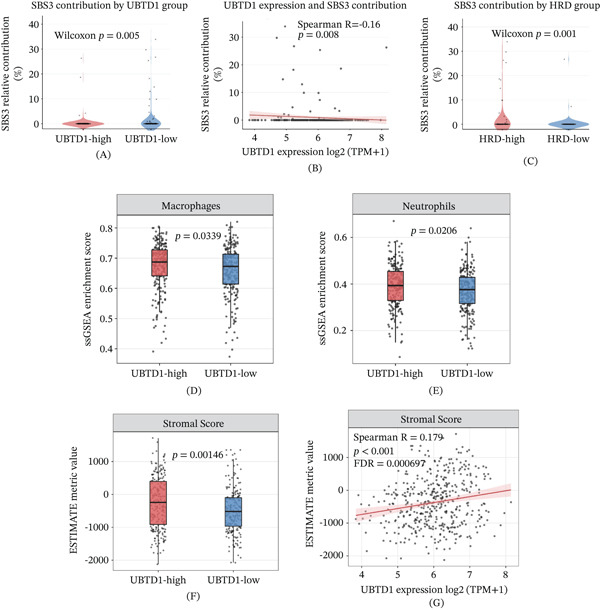
UBTD1 expression is associated with SBS3 mutational signature and stromal‐immune remodeling in ovarian cancer. (A) Relative contribution of the HRD‐related SBS3 mutational signature in UBTD1‐high (*n* = 141) and UBTD1‐low (*n* = 142) tumors. (B) Spearman correlation between continuous UBTD1 expression and SBS3 contribution. (C) SBS3 contribution in HRD‐low (*n* = 81) and HRD‐high (*n* = 85) tumors. HRD‐high tumors showed higher SBS3 contribution. (D–E) (D) ssGSEA‐based macrophages and (E) neutrophils enrichment scores in UBTD1‐high (*n* = 141) and UBTD1‐low (*n* = 143) tumors. (F) ESTIMATE‐derived stromal scores in UBTD1‐high (*n* = 141) and UBTD1‐low (*n* = 143) tumors. (G) Spearman correlation between UBTD1 expression and stromal score. Group comparisons were performed using the Wilcoxon rank‐sum test.

Because UBTD1‐low tumors showed higher HRD scores, we next assessed SBS mutational signatures in TCGA‐OV. SBS3, a homologous recombination deficiency‐related signature, was more enriched in UBTD1‐low tumors than in UBTD1‐high tumors (Figure 3A). UBTD1 expression was modestly but significantly negatively correlated with SBS3 contribution (Figure 3B). As expected, SBS3 contribution was also higher in HRD‐high tumors than in HRD‐low tumors (Figure 3C). These results further link low UBTD1 expression to HRD‐related mutational features in ovarian cancer.

Given the recognized interplay between genomic alterations and the tumor microenvironment, we next evaluated immune and stromal features associated with UBTD1 expression. ssGSEA‐based immune infiltration analysis revealed modestly increased macrophage and neutrophil infiltration scores in the UBTD1‐high group (Figure 3D–E), whereas other immune cell populations showed no significant differences (Figure S3). Furthermore, ESTIMATE analysis demonstrated significantly elevated stromal scores in UBTD1‐high tumors compared with UBTD1‐low tumors (Figure 3F), and UBTD1 expression was positively correlated with stromal score across TCGA‐OV samples (Figure 3G), indicating enhanced stromal enrichment.

Together, these findings indicate that UBTD1‐low tumors are enriched for HRD and SBS3‐related mutation signatures, whereas UBTD1‐high tumors are characterized by a more stromal‐rich microenvironment with selected immune‐cell enrichment.

### 3.4. UBTD1 Is Highly Expressed in Ovarian Cancer and Correlates With Poor Prognosis

**Figure 4:**
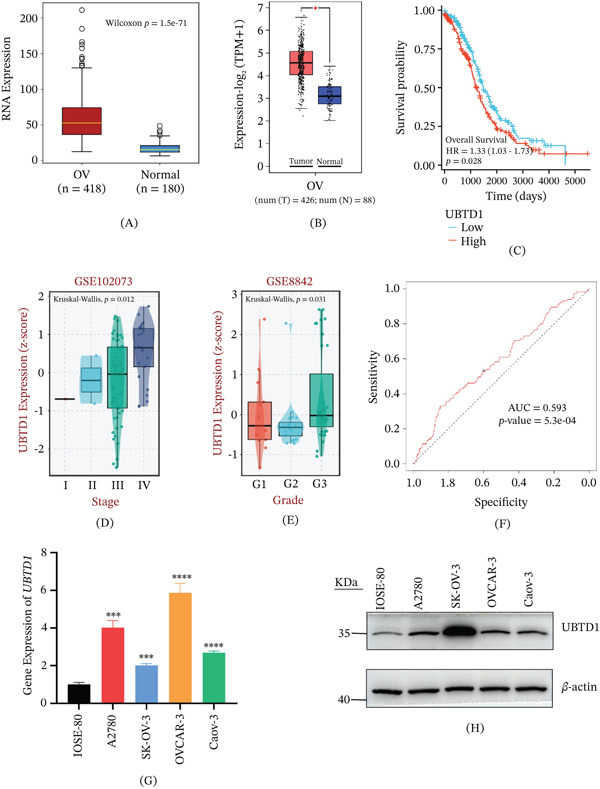
UBTD1 is highly expressed in ovarian cancer and correlates with poor prognosis. (A) Analysis of UBTD1 mRNA expression levels in normal tissues (*n* = 180) and tumor tissues (*n* = 418) from the OncoDB website. Wilcoxon *p* = 1.5 × 10^−71^. (B) The GEPIA2 website analyzes the mRNA expression levels of UBTD1 in normal tissues (*n* = 88) and tumor tissues (*n* = 426). *p* < 0.05. (C) Kaplan–Meier survival analysis reveals the relationship between UBTD1 expression level and overall survival in ovarian cancer patients (*n* = 379; low = 188, high = 191). Patients were stratified into high‐ and low‐expression groups based on the median expression level (median cutoff) of UBTD1. *p* value was determined by the log‐rank test. HR, hazard ratio. (D–E) The relationship between (D) FIGO staging (GSE102073; *n* = 96) and (E) histological grading (GSE8842; *n* = 83) of ovarian cancer patients and UBTD1 expression levels. *p* value was determined by the Kruskal–Wallis test. (F) The ROC curve for UBTD1 as a diagnostic biomarker for ovarian cancer patients (*n* = 1347), with an AUC of 0.593 (*p* = 5.3 × 10^−4^). (G–H) UBTD1 exhibited high expression in ovarian cancer cell lines at both the (G) mRNA and (H) protein levels, *β*‐actin served as a loading control. *N* = 3; means ± SD, ∗∗∗*p* < 0.001; ∗∗∗∗*p* < 0.0001; *T*‐test.

To assess the diagnostic potential of UBTD1 in ovarian cancer, we integrated bioinformatic and experimental analyses. Differential expression analysis indicated a significant upregulation of UBTD1 between ovarian cancer samples and the corresponding adjacent noncancerous tissues (Figure 4A,B). Kaplan–Meier survival analysis indicated that elevated UBTD1 expression was associated with reduced overall survival (Figure 4C). Additionally, UBTD1 was positively correlated with both advanced FIGO stage and elevated histological grade (Figure 4D,E). The ROC curve analysis demonstrated an AUC of 0.593 (*p* < 0.05), suggesting that UBTD1 has limited but potentially informative diagnostic value in ovarian cancer (Figure 4F).

Experimental validation demonstrated a significant increase of UBTD1 at both mRNA and protein levels in ovarian cancer cell lines compared to IOSE‐80, as assessed by qRT‐PCR and immunoblotting (Figure 4G,H). UBTD1 is overexpressed in ovarian cancer, associated with aggressive clinicopathological features and reduced patient survival. These findings support its potential role as an oncogene and provide a basis for further functional characterization.

### 3.5. Knockdown of UBTD1 Inhibits Proliferation of Ovarian Cancer Cells

**Figure 5:**
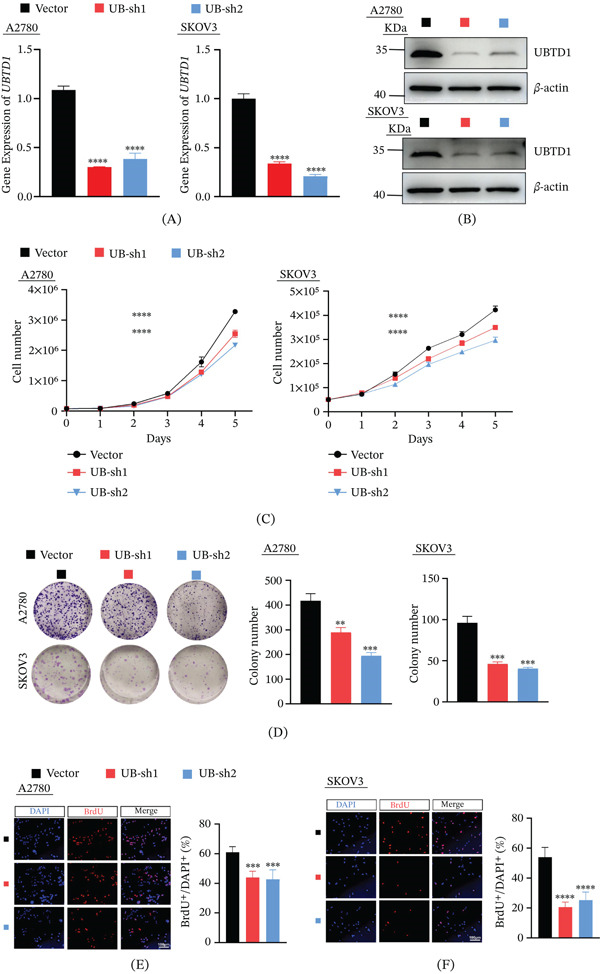
Knockdown of UBTD1 inhibits proliferation of ovarian cancer cells. (A–B) Assessment of UBTD1 knockdown efficiency in A2780 and SK‐OV‐3 cell lines was conducted, with (A) representing mRNA levels and (B) representing protein levels, *n* = 3. (C) Growth curve analysis of ovarian cancer cells over 5 days (*n* = 3). (D) The clonogenic potential of A2780 and SK‐OV‐3 cells was assessed by colony formation assay, and the results were quantified and statistically analyzed (*n* = 3). (E–F) BrdU incorporation assays in (E) A2780 and (F) SK‐OV‐3 cells, showing representative immunofluorescence images and quantitative results (*n* = 5). Scale bar = 100 *μ*m. Means ± SD, ∗∗*p* < 0.01; ∗∗∗*p* < 0.001; ∗∗∗∗*p* < 0.0001; *T*‐test. Vector = control shRNA; UB − sh1 = UBTD1 shRNA1; UB − sh2 = UBTD1 shRNA2.

We developed stable UBTD1‐knockdown models in A2780 and SK‐OV‐3 cell lines to investigate UBTD1′s biological role in ovarian cancer. Knockdown efficiency was confirmed by qRT‐PCR for transcript expression and by immunoblotting for protein abundance (Figure 5A,B). Subsequently, multiple functional assays were performed to examine how UBTD1 influences malignant phenotypes. Specifically, UBTD1 knockdown reduced the cellular proliferation rate (Figure 5C), suppressed colony formation capacity (Figure 5D), and decreased DNA synthesis activity as measured by BrdU incorporation (Figure 5E,F). These findings indicate that UBTD1 enhances proliferation and clonogenicity in ovarian cancer cells.

### 3.6. Knockdown of UBTD1 Induces G0/G1 Phase Arrest in Ovarian Cancer Cells by Modulating Cell Cycle Regulators

**Figure 6:**
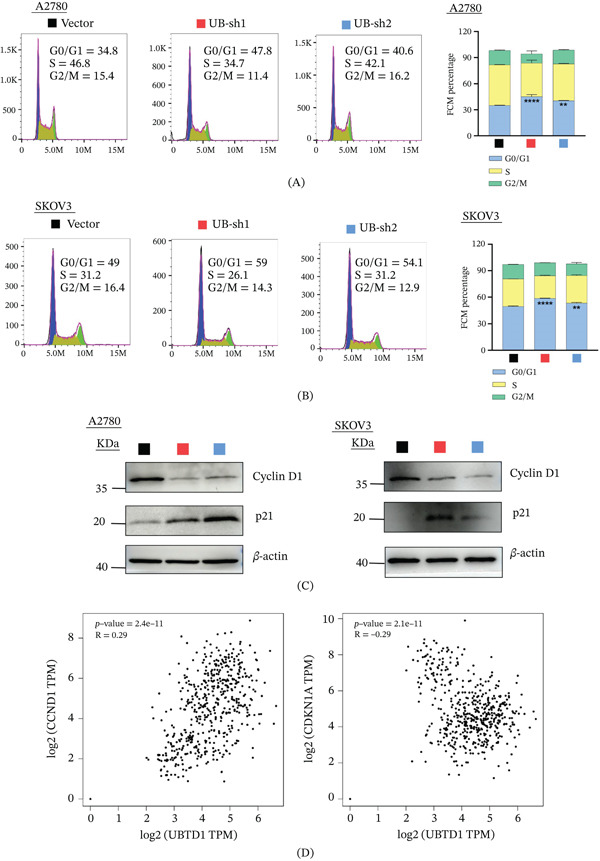
Knockdown of UBTD1 induces G0/G1 phase arrest in ovarian cancer cells by modulating cell cycle regulators. (A–B) FACS analysis of cell cycle distribution following UBTD1 knockdown in (A) A2780 and (B) SK‐OV‐3 cell lines, with statistical results shown (*n* = 3). Means ± SD, ∗∗*p* < 0.01; ∗∗∗∗*p* < 0.0001; *T*‐test. (C) Immunoblot analysis of G0/G1‐phase markers (Cyclin D1 and p21) following UBTD1 knockdown. (D) The correlation between UBTD1 and G0/G1‐phase–related genes (CCND1 and CDKN1A/p21) at the mRNA level was evaluated utilizing the GEPIA2 database. *p* value was determined by the hypothesis testing. R is the Pearson correlation coefficient. ∗∗∗∗*p* < 0.0001.

We conducted cell cycle analysis to explore the mechanism responsible for the decreased proliferation. Flow cytometric analysis showed that UBTD1 knockdown led to a marked accumulation of cells in G0/G1 phase (Figure 6A,B). Correspondingly, western blot revealed that UBTD1 knockdown upregulated the cell cycle inhibitor p21 and downregulated the G1/S phase driver cyclin D1 (Figure 6C). Analysis of patient data from the GEPIA2 database confirmed the clinical significance of these findings. UBTD1 expression showed a significant negative correlation with p21 expression and a positive correlation with cyclin D1 levels (Figure 6D). The findings indicate that UBTD1 promotes ovarian cancer cell proliferation by aiding the G1/S phase transition. The consistency between experimental and clinical data underscores its role as an oncogene.

### 3.7. UBTD1 Knockdown Inhibits Ovarian Cancer Cell Migration and Invasion and Is Accompanied by EMT‐Related Changes

**Figure 7:**
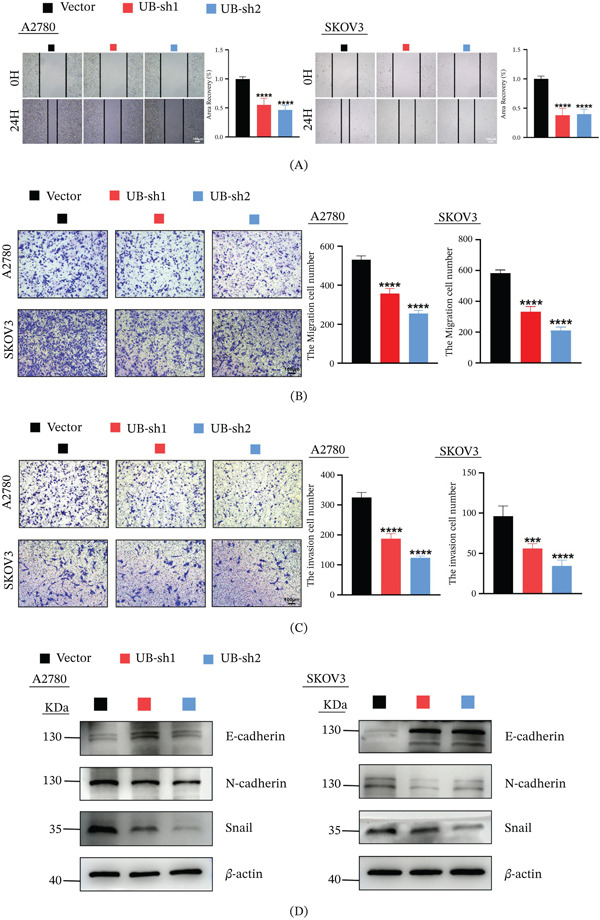
UBTD1 knockdown inhibits ovarian cancer cell migration and invasion and is accompanied by EMT‐related changes. (A) Wound healing assay indicating the migration ability of A2780 and SK‐OV‐3 cells following UBTD1 knockdown, with quantitative statistics presented (*n* = 3). (B) Transwell detection results of ovarian cancer cells, including representative images of crystal violet stained cells migrating through the membrane and statistical quantification results for each group (*n* = 3). (C) A Matrigel invasion assay was performed to compare control and UBTD1 knockdown cells. The invaded cells were quantified from multiple fields (*n* = 3). (D) Immunoblots to detect the protein expression of the key regulators involved in cell migration, including E‐cadherin, N‐cadherin, and Snail following UBTD1 knockdown in indicated cells. Scale bar = 100 *μ*m. Means ± SD, ∗∗∗*p* < 0.001; ∗∗∗∗*p* < 0.0001; *T*‐test.

Following the observed effect on proliferation, we evaluated the role of UBTD1 in cell motility. Wound healing (Figure 7A) and transwell migration assays (Figure 7B) collectively demonstrated that UBTD1 knockdown potently suppressed the migration of ovarian cancer cells. Moreover, its knockdown also markedly reduced cellular invasion in a matrigel‐based assay (Figure 7C).

To investigate whether EMT‐associated changes were involved, we assessed the expression of several representative EMT markers. Immunoblotting analysis revealed that UBTD1 knockdown was associated with elevated E‐cadherin expression and reduced levels of N‐cadherin and Snail (Figure 7D). These observations indicate that UBTD1 may contribute to a mesenchymal‐like and invasive phenotype in ovarian cancer cells.

### 3.8. UBTD1 Knockdown Sensitizes Ovarian Cancer Cells to Cisplatin by Promoting Apoptosis

**Figure 8:**
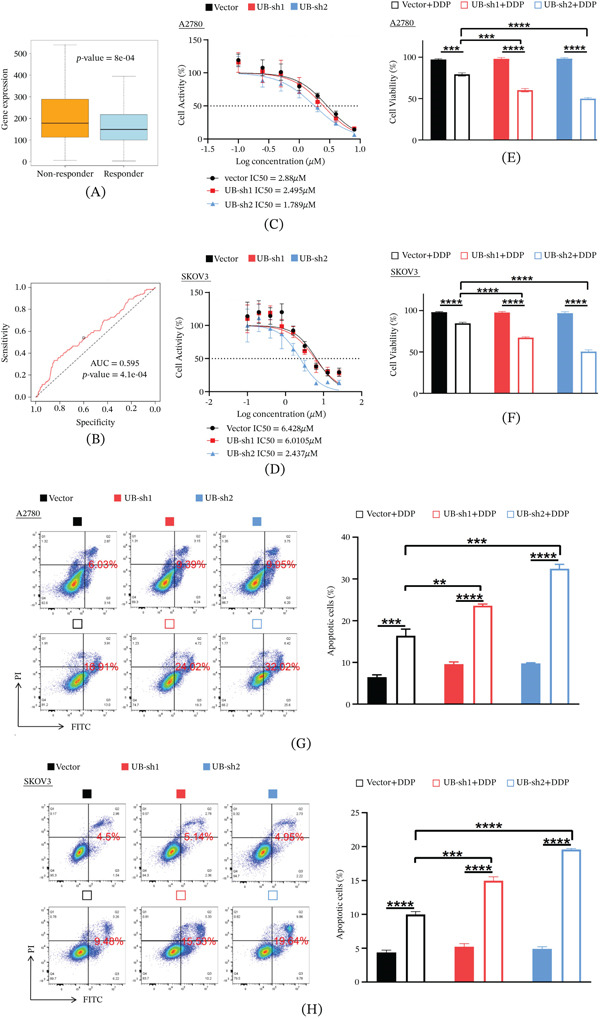
UBTD1 knockdown sensitizes ovarian cancer cells to cisplatin by promoting apoptosis. (A) Comparison of UBTD1 mRNA expression levels between cisplatin‐responsive (*n* = 1095) and nonresponsive (*n* = 114) patients. *p* = 8 × 10^−4^; *T*‐test. (B) Predictive value of UBTD1 expression for cisplatin response status (*n* = 1209), with an AUC of 0.595 (*p* = 4.1 × 10^−4^). (C–D) The dose‐response curve and calculated half‐maximal inhibitory concentration values of cisplatin (DDP) showed drug sensitivity in UBTD1 knockdown (C) A2780 and (D) SK‐OV‐3 cells (*n* = 3). (E–F) Add DDP treatment to (E) A2780 and (F) SK‐OV‐3 cells with UBTD1 gene knockdown for 72 h to detect cell viability (*n* = 3). (G–H) Apoptosis induced by DDP was assessed by flow cytometry in (G) A2780 and (H) SK‐OV‐3 cells following UBTD1 knockdown (*n* = 3). Means ± SD, ∗∗*p* < 0.01; ∗∗∗*p* < 0.001; ∗∗∗∗*p* < 0.0001; *T*‐test.

Platinum resistance remains a formidable therapeutic challenge in advanced ovarian cancer. Given our previous findings that UBTD1 promotes malignant progression, we proposed that it might also regulate cellular response to cisplatin. We evaluated UBTD1 gene expression levels in tumor tissues and compared its levels between patients with and without cisplatin response. The study found that median UBTD1 expression was elevated in patients who did not respond to treatment compared with those who did (Figure 8A). ROC analysis suggested a limited potential for UBTD1 expression to distinguish between cisplatin‐responsive and nonresponsive cases (Figure 8B).

To test this, we examined the half‐maximal inhibitory concentration (IC_50_) of cisplatin following UBTD1 knockdown. CCK‐8 assays showed that UBTD1 knockdown significantly decreased the IC_50_ in both A2780 (Figure 8C) and SK‐OV‐3 (Figure 8D) cell lines, indicating enhanced drug sensitivity. Consistent with the IC_50_ results, UBTD1‐knockdown cells exhibited markedly lower viability than controls when treated with their respective IC_50_ concentrations (Figure 8E,F), demonstrating that UBTD1 silencing not only reduces the required inhibitory drug concentration but also potentiates cisplatin cytotoxicity. We investigated the underlying mechanism by analyzing apoptosis through Annexin V‐FITC/PI double staining. Although no significant difference in baseline apoptosis was observed between groups under untreated conditions, UBTD1‐knockdown cells showed a markedly increased apoptosis rate following IC_50_ cisplatin treatment (Figure 8G,H). This suggests that UBTD1 mediated enhancement of cisplatin sensitivity occurs, at least partially, through promotion of drug‐induced apoptosis. Taken together, the data support a potential role for UBTD1 in modulating cisplatin response in ovarian cancer cells.

### 3.9. UBTD1 Is Associated With TNF/AP‐1–Related Signaling Changes in Ovarian Cancer Cells

**Figure 9:**
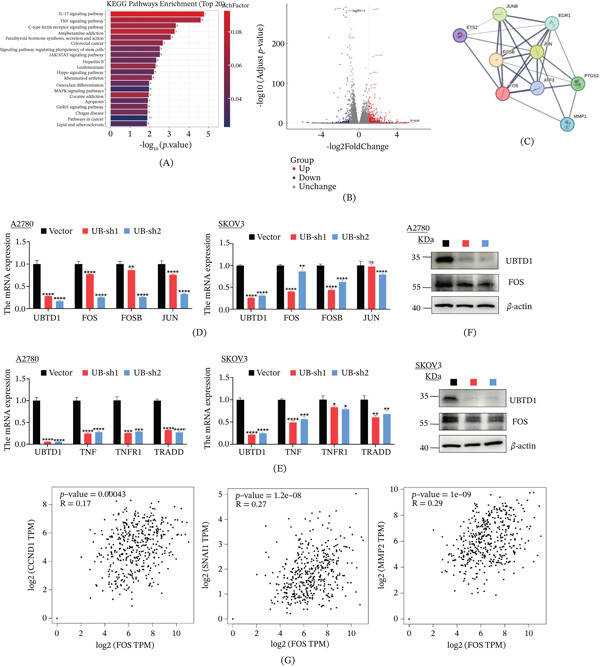
UBTD1 is associated with TNF/AP‐1–related signaling changes in ovarian cancer cells. (A) KEGG pathway enrichment analysis results (*n* = 3). (B) Volcanic diagram displays differentially expressed genes between the control group and the UBTD1 knockdown group (*n* = 3). (C) PPI network of the differentially expressed genes. (D) Detection of expression levels of FOS, FOSB, and JUN in A2780 and SK‐OV‐3 cells after UBTD1 gene knockdown by qRT‐PCR (*n* = 3). (E) qRT‐PCR was performed to examine the expression of key molecules in the TNF signaling pathway following UBTD1 knockdown (*n* = 3). (F) Immunoblot detection of FOS protein levels in A2780 and SK‐OV‐3 cells following UBTD1 knockdown. (G) GEPIA2 correlation analysis was performed in ovarian cancer to examine the relationship between FOS expression and relevant genes, including cell cycle regulators such as CCND1, as well as EMT markers like SNAI1 and MMP2. Data are analyzed by (D and E) *T*‐test and (G) hypothesis test, and means ± SD, ∗*p* < 0.05; ∗∗*p* < 0.01; ∗∗∗*p* < 0.001; ∗∗∗∗*p* < 0.0001; ns, no significance. R is the Pearson correlation coefficient.

To identify the molecular mechanism underlying the oncogenic role of UBTD1, we conducted transcriptome sequencing on control and UBTD1‐knockdown A2780 cells. KEGG enrichment analysis indicated significant suppression of the TNF signaling pathway following UBTD1 knockdown (Figure 9A). Among the differentially expressed genes (DEGs), the transcription factors FOS and FOSB were notably downregulated (Figure 9B), and protein–protein interaction (PPI) analysis further highlighted marked enrichment in nodes of the AP‐1 complex, including core components such as FOS, FOSB, and JUN (Figure 9C), indicating that the AP‐1 complex may be a downstream effector of UBTD1.

We validated these findings in A2780 and SK‐OV‐3 cell lines. The qRT‐PCR validation demonstrated that UBTD1 knockdown significantly decreased mRNA levels of FOS, FOSB, and key TNF pathway molecules, aligning with transcriptomic findings (Figures 9D,E). Meanwhile, we also verified the downregulation of FOS protein levels (Figure 9F). Correlation analysis indicated a positive association between FOS expression and cell cycle–related genes (CCND1) as well as EMT markers (SNAI1 and MMP2), highlighting AP‐1′s pivotal role in controlling proliferation and metastasis (Figure 9G).

These findings indicate that UBTD1 may promote ovarian cancer progression in association with TNF/AP‐1 signaling.

### 3.10. FOS Overexpression Partially Reverses the Tumor‐Suppressive Effects of UBTD1 Knockdown

**Figure 10:**
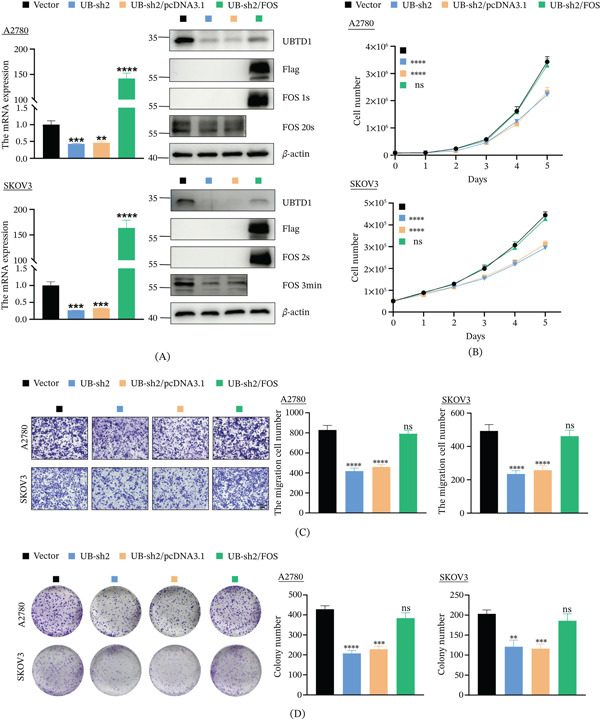
FOS overexpression partially reverses the tumor‐suppressive effects of UBTD1 knockdown. (A) Validation of FOS overexpression in UBTD1 knockdown cells by qRT‐PCR and immunoblot. (B) A 5‐day growth curve analysis was performed to assess the proliferative capacity of A2780 and SK‐OV‐3 ovarian cancer cells in which FOS was overexpressed against a background of UBTD1 knockdown (*n* = 3). (C) In vitro migration assay in A2780 and SK‐OV‐3 cells to assess the effect of FOS overexpression in a UBTD1‐knockdown background, with quantitative analysis shown (*n* = 3). (D) To assess the effect of FOS overexpression, A2780 and SK‐OV‐3 cells under UBTD1 knockdown conditions were subjected to colony formation assays, with results quantified (*n* = 3). Data were analyzed by (B) two‐way ANOVA and (C and D) *T*‐test, and means ± SD, ∗*p* < 0.05; ∗∗*p* < 0.01; ∗∗∗*p* < 0.001; ∗∗∗∗*p* < 0.0001; ns, no significance. UB‐sh2/pcDNA3.1 = UBTD1 shRNA2 + pcDNA3.1 vector control; UB‐sh2/FOS = UBTD1 shRNA2 + FOS overexpression.

To determine whether the AP‐1 complex mediates the oncogenic function of UBTD1, we carried out rescue assays in UBTD1‐knockdown cells by overexpressing FOS. FOS overexpression was efficiently validated at both mRNA and protein levels (Figure 10A). A series of functional assays demonstrated that FOS overexpression partially rescued the malignant phenotypes suppressed by UBTD1 knockdown. Specifically, FOS restoration recovered cell proliferation to near‐control levels (Figure 10B), largely reversed the impairment in cell migration (Figure 10C), and significantly restored colony forming ability (Figure 10D). The evidence indicates that UBTD1 may promote ovarian cancer cell proliferation, migration, and invasion, at least in part through AP‐1–related transcriptional regulation, with FOS likely acting as one of the downstream mediators. Together, the data support a functional link between UBTD1 and FOS in ovarian cancer progression.

## 4. Discussion

Among gynecological malignancies, ovarian cancer remains one of the leading causes of cancer‐related death [3, 23, 24]; largely because many cases are diagnosed at an advanced stage and later develop treatment resistance [25–30]. These challenges make it especially important to identify more reliable prognostic indicators and potential targets for therapeutic intervention. In this study, we found that UBTD1 is associated with both mutation‐related genomic features and malignant progression in ovarian cancer. UBTD1 expression distinguished different tumor states, whereas functional experiments showed that UBTD1 was upregulated in ovarian cancer and that its knockdown suppressed malignant phenotypes and cisplatin resistance.

Our integrative analyses suggest that UBTD1 expression is associated with mutation‐related genomic features and the tumor microenvironment in ovarian cancer. At the single‐cell level, UBTD1‐low/virtual knockdown–like malignant cells showed increased CopyKAT‐inferred CNV burden and higher nucleotide excision repair and cell cycle checkpoint scores, suggesting a malignant‐cell state related to mutation‐associated genomic alterations. Consistently, TCGA‐OV analysis showed that UBTD1‐low tumors had higher tumor mutation burden, increased homologous recombination and DNA repair pathway activity, elevated HRD scores, and greater SBS3 mutational signature contribution. These findings indicate that reduced UBTD1 expression is associated with HRD/SBS3–related mutation features rather than a change driven by one specific mutated gene. By comparison, UBTD1‐high tumors showed higher stromal scores and modest enrichment of macrophages and neutrophils, suggesting a relationship between UBTD1 expression and stromal‐immune microenvironment remodeling.

The UBTD family, characterized by ubiquitin‐like domains, is known to regulate protein degradation and cell cycle. UBTD1 has been proposed as a diagnostic marker in multiple cancer types, although its biological function seems to vary depending on the tumor context. In colorectal cancer, UBTD1 forms a complex with the E3 ligase *β*‐TrCP, enhancing c‐Myc ubiquitination and preventing its proteasomal degradation. This stabilization of c‐Myc activates downstream glycolytic enzymes, such as HK2, promoting metabolic reprogramming and malignant phenotypes [11]. In contrast, UBTD1 appears to act as a tumor suppressor in certain contexts, such as prostate and lung cancers, via modulation of YAP degradation [18]. In gastric cancer, UBTD1 promotes the degradation of the E3 ligase MDM2 via a ubiquitin‐dependent mechanism, stabilizing p53 and inducing cellular senescence. Activated p53 further upregulates UBTD1 transcription, forming a positive feedback loop that amplifies tumor‐suppressive signaling, whereas low UBTD1 expression disrupts this loop, impairing p53 function and contributing to malignancy and poor prognosis [31]. Our data consistently support a tumor‐promoting role for UBTD1 in ovarian cancer. This is evidenced by its high expression, association with poor prognosis, and the multifaceted malignant phenotypes it regulates.

To delineate the downstream mechanism of UBTD1, RNA sequencing was performed in A2780 cells. The study found that UBTD1 knockdown significantly reduced the expression of FOS and FOSB, with DEGs showing enrichment in the AP‐1 transcription factor complex. KEGG enrichment analysis also indicated that genes involved in TNF signaling were significantly downregulated. This pathway exerts two distinct effects in tumors: It can activate the apoptosis pathway to exert antitumor effects [32–36], yet in the tumor microenvironment, persistent low‐level TNF production by tumor and other cells may induce chronic inflammation and immune suppression by activating downstream pathways like NF‐*κ*B, thus promoting tumor development and progression [37, 38]. AP‐1, a dimeric complex of FOS, JUN, and ATF family members, acts as a rapid cellular response factor to external stimuli and plays central roles in proliferation, differentiation, apoptosis, and inflammation [39–42]. It integrates stress and proliferative signals, contributing critically to tumor development [43–47]. In endometrial cancer, AP‐1 collaborates with SF1 and LRH1 to promote cell cycle progression [48], whereas in head and neck squamous cell carcinoma, increased c‐Fos expression boosts EMT and cancer stemness markers [49].

To functionally validate the link between UBTD1 and AP‐1, we performed rescue experiments, showing that FOS overexpression restored proliferation, colony formation, and migration in UBTD1‐knockdown cells, supporting the possibility that FOS may function as a downstream mediator of UBTD1. Moreover, correlation analysis indicated that FOS expression positively correlates with cell cycle genes (CCND1 and CDKN1A) and EMT markers (SNAI1 and MMP2), consistent with a role in regulating proliferation and metastasis [50, 51]. These data support a link between UBTD1 and TNF/AP‐1–related signaling in OC and suggest that this pathway may contribute to the regulation of genes involved in cell cycle progression and EMT‐related changes.

In summary, this study suggests that UBTD1 has a multifaceted role in ovarian cancer. UBTD1‐low tumors were characterized by mutation‐related genomic features, including increased inferred CNV burden, higher TMB, elevated HRD scores, and SBS3 mutational signature enrichment. UBTD1‐high tumors were associated with stromal enrichment and selected immune‐cell infiltration. Functional experiments further demonstrated that UBTD1 promotes malignant phenotypes and cisplatin resistance, at least partly through TNF/AP‐1/FOS–related signaling. These findings provide a basis for further investigation of UBTD1 as a potential marker connecting mutation‐related genomic alterations, tumor microenvironment features, and ovarian cancer progression.

Although this study offers valuable insights, a number of limitations remain. Although our analyses revealed associations among UBTD1 expression, genomic instability, and stromal/immune microenvironment characteristics, the present study does not establish direct mechanistic links between these processes. Additional studies are required to determine whether UBTD1 directly contributes to the interaction between genomic alterations and tumor microenvironment remodeling in ovarian cancer. The relationship between UBTD1 expression and HRD/SBS3–related mutation patterns remains an association that requires further mechanistic validation. Although the in vitro systems used in this study provided useful information, they are still unable to fully reflect the multifactorial in vivo tumor milieu. Future studies will be aimed at validating the functions of UBTD1 using subcutaneous xenograft models in BALB/c nude mice and orthotopic ovarian cancer models in C57BL/6 mice, as these systems can better recapitulate in vivo tumor growth and progression. Furthermore, the lack of clinical sample validation represents another limitation of this study. Therefore, future investigations will also include the collection and analysis of clinical ovarian cancer specimens to further verify the clinical relevance of our findings. Additionally, the specific downstream targets of AP‐1 in this context warrant further investigation, and their relevance in vivo will be critical for clinical translation. Future research will be aimed at clarifying the molecular mechanisms downstream of UBTD1 and developing targeted inhibitors to advance new therapeutic strategies for ovarian cancer.

To date, there are no specific targeted drugs or inhibitors that directly target UBTD1, nor are there any clinical trials registered with UBTD1 as the primary therapeutic target. Although several studies have revealed mechanistic interactions between UBTD1 and oncogenic pathways, these findings have not yet translated into UBTD1‐specific therapeutics. Therefore, future work is required to explore both direct and indirect therapeutic strategies that involve UBTD1 or its downstream effectors in ovarian cancer and other malignancies.

## 5. Conclusions

This study indicates that UBTD1 is associated with mutation‐related genomic alterations and stromal‐immune remodeling in ovarian cancer. UBTD1‐low tumors showed higher mutation burden, HRD‐related features, SBS3 mutational signature contribution, and inferred copy‐number alteration burden, whereas UBTD1‐high tumors were linked to stromal enrichment and selected immune‐cell infiltration. Functional assays further demonstrated that UBTD1 promotes ovarian cancer proliferation, migration, invasion, and cisplatin resistance, potentially through TNF/AP‐1/FOS–related signaling. These findings suggest that UBTD1 may serve as a multifunctional regulator connecting genomic alteration, tumor microenvironment remodeling, and malignant progression in ovarian cancer.

## Author Contributions

Cuiping Yang, Yongbin Chen, and Baiyang Liu initiated the study and drafted the manuscript. Aixin Liu performed the cell experiments. Yanxia Chen supervised the experimental process. Xian Zhao, Junying Zhou, and Guanying Feng conducted bioinformatics analyses and literature surveys. Huijuan Zhang provided thoughtful suggestions. The article has been read and approved by all authors.

## Funding

This study was supported by the National Natural Science Foundation of China (82173110) and the Eastern Talent Plan Leading Project 2024 (BJWS2024079).

## Disclosure

All authors contributed to this article and approved the submitted version.

## Ethics Statement

The authors have nothing to report.

## Conflicts of Interest

The authors declare no conflicts of interest.

## Supporting Information

Additional supporting information can be found online in the Supporting Information section.

## Supporting information


**Supporting Information 1** The supporting figures file contains Figures S1–S3, showing additional single‐cell analyses, CopyKAT‐inferred CNV and hdWGCNA results, TCGA‐OV mutation spectrum analyses, and ssGSEA‐based immune infiltration analyses stratified by UBTD1 expression.

**Supporting Information 2** The supporting table file contains the antibodies used in the experiments and the primer sequences used for qRT‐PCR.

## Data Availability

The data that support the findings of this study are openly available in NCBI Gene Expression Omnibus at https://www.ncbi.nlm.nih.gov/geo/query/acc.cgi?acc=GSE325761, Reference Number: GSE325761.
