# Supplementary material for: UBTD1 Drives Ovarian Cancer Progression via Mutation‐Associated Alterations, Stromal Microenvironment Remodeling, and TNF/AP‐1 Signaling
Source: Hum Mutat. 2026 Jul 22;2026:9479429. doi: 10.1155/humu/9479429 (PMC13390016; doi:10.1155/humu/9479429)
Supplement: Supplementary file 1 — Supporting Information 1 The supporting figures file contains Figures S1–S3, showing additional single‐cell analyses, CopyKAT‐inferred CNV and hdWGCNA results, TCGA‐OV mutation spectrum analyses, and ssGSEA‐based immune infiltration analyses stratified by UBTD1 expression. [file HUMU-2026-9479429-s001.docx]

**Supplementary Figures**

**
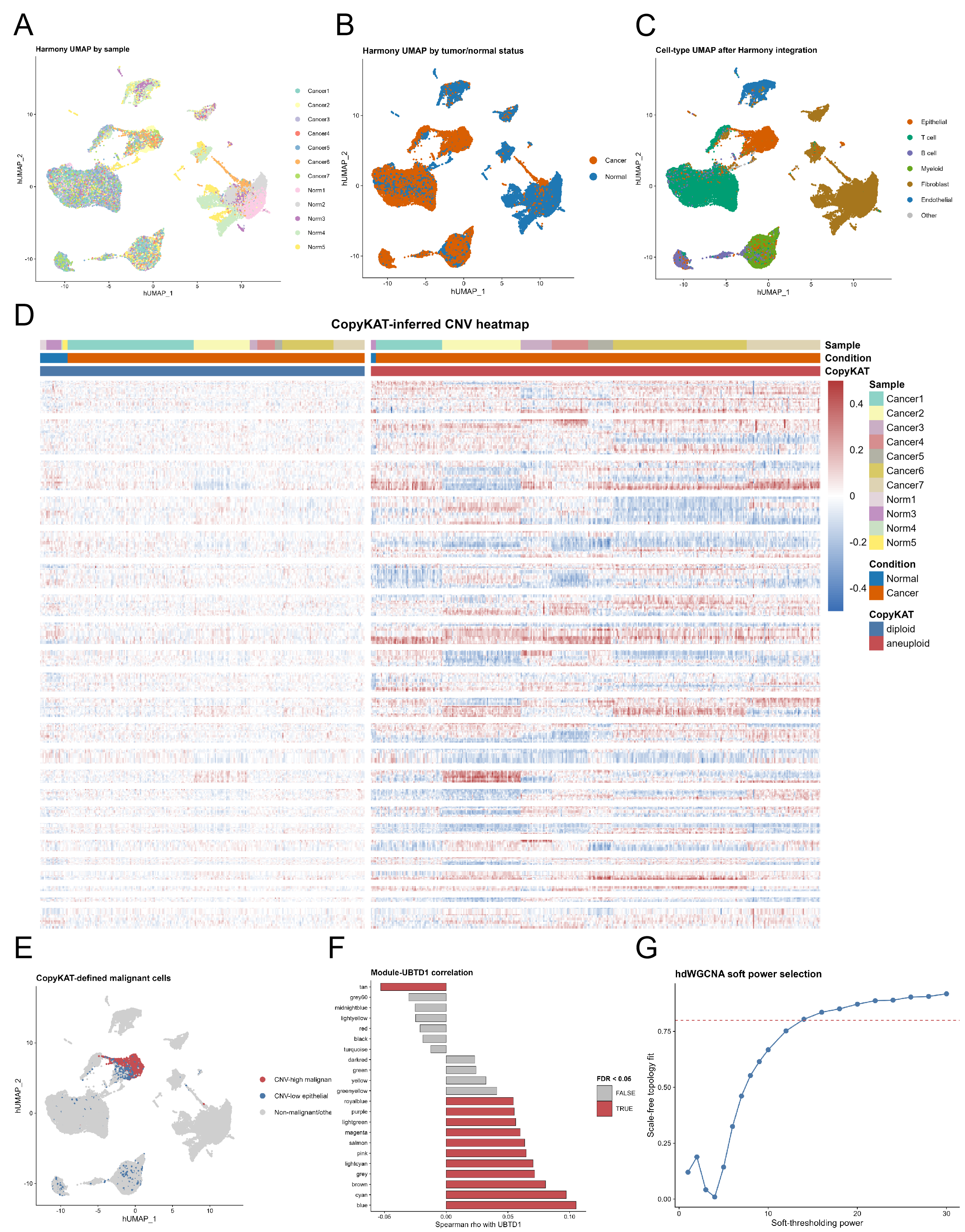
**

Figure S1. Identification of malignant cells, UBTD1 expression pattern, and hdWGCNA network construction in ovarian cancer single-cell RNA-seq data (GSE184880).

1. Harmony-integrated UMAP colored by sample origin. GSE184880 were processed using Seurat and integrated with Harmony to remove sample-specific batch effects. Each dot represents one single cell, and different colors indicate individual cancer or normal samples. (B) Harmony-integrated UMAP colored by tumor or normal status. (C) Cell-type annotation on integrated UMAP based on canonical marker genes, including epithelial cells, T cells, B cells, myeloid cells, fibroblasts, endothelial cells and other cell populations. (D) CopyKAT-inferred copy number variation heatmap. Columns represent genomic regions, rows represent single cells. The upper annotation bars indicate sample origin, tumor/normal condition, and CopyKAT prediction. Red indicates copy number gains, and blue indicates copy number losses. (E) UMAP of CopyKAT-defined malignant cells. CNV-high/aneuploid cells were defined as malignant cells, while CNV-low epithelial cells and other cell types were annotated as non-malignant/other cells. In total, 2,636 malignant cells were verified by CopyKAT. 670 UBTD1-positive and 1966 UBTD1-negative cells. (F) Spearman’s correlation between hdWGCNA module eigengenes and UBTD1 expression in malignant cells. Red bars indicate modules significant after false discovery rate (FDR) correction; gray bars indicate non-significant modules. (G) Soft-thresholding power selection for hdWGCNA. The scale-free topology fit index was calculated across different powers. The dashed horizontal line represents the fitting cutoff, and the selected power was used to construct the weighted gene co-expression network.


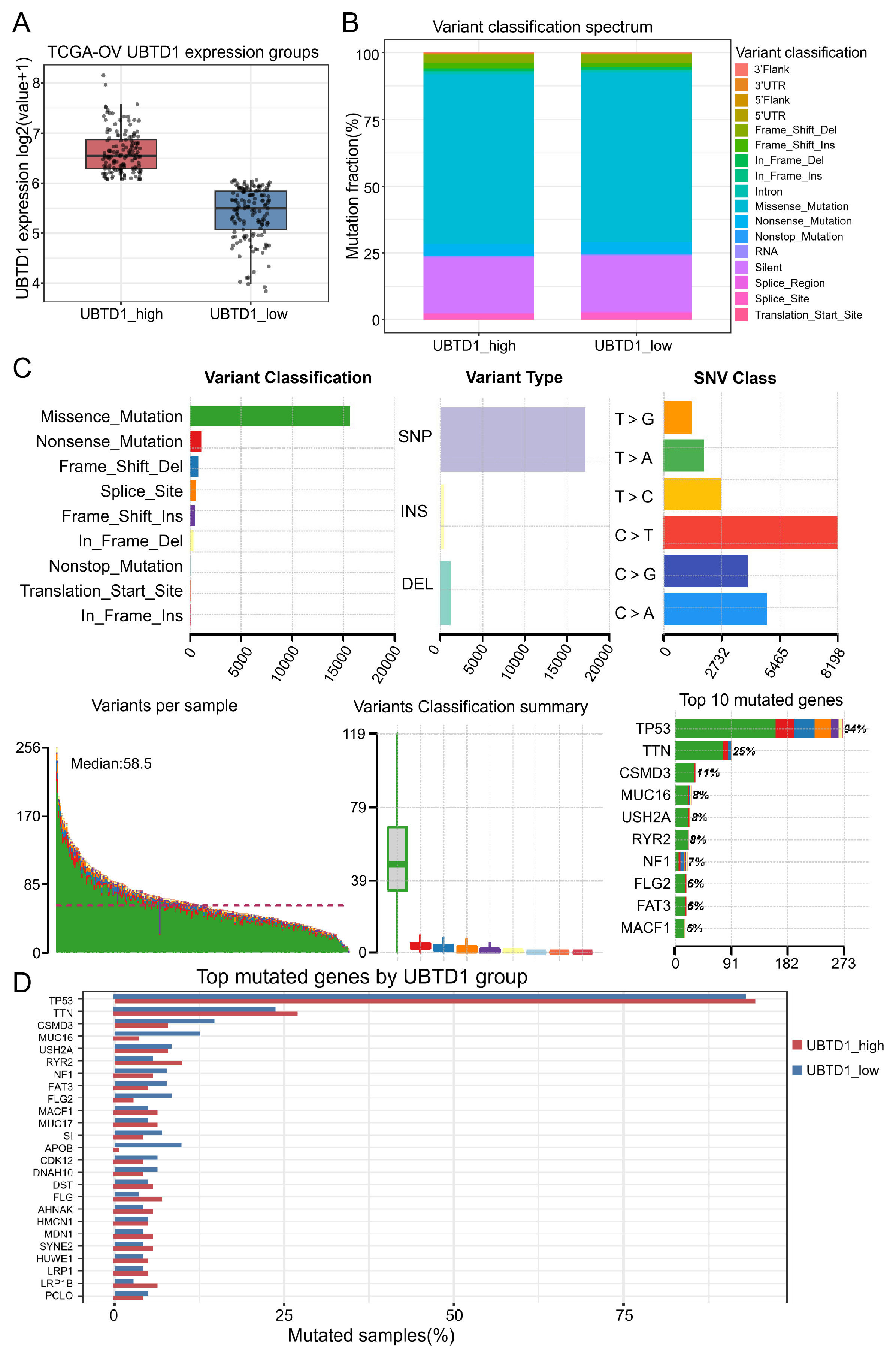


Figure S2. Comparison of mutation spectrum between UBTD1-high and UBTD1-low groups in TCGA-OV cohort. (A) Boxplot of UBTD1 expression across the two subgroups. TCGA-OV samples (n=284) were divided into UBTD1-high (n=141) and UBTD1-low (n=143) groups using the median expression as a cutoff. UBTD1 expression levels are presented as \(\log_2(\text{TPM}+1)\). Each dot corresponds to an individual tumor sample. (B) Distribution of somatic variant classification in UBTD1-high and UBTD1-low groups. Each stacked bar represents the proportion of somatic mutations with distinct functional impacts. Color-coded mutation categories include Missense_Mutation, Nonsense_Mutation, Frame_Shift_Del/Ins, Intron, Splice_Site/Region, Silent, RNA, and other categories. (C) Global mutation landscape of TCGA-OV cohort (n=284), consisting of six panels: variant type, SNV class, variant classification summary, top mutated genes, number of variants per sample (median=59.5), and overall tumor mutation burden summary. (D) The top 25 recurrently mutated genes compared between UBTD1-high and UBTD1-low groups. Horizontal bars illustrate the proportion of samples carrying mutations in each gene. Red bars indicate UBTD1-high samples, and blue bars indicate UBTD1-low samples.


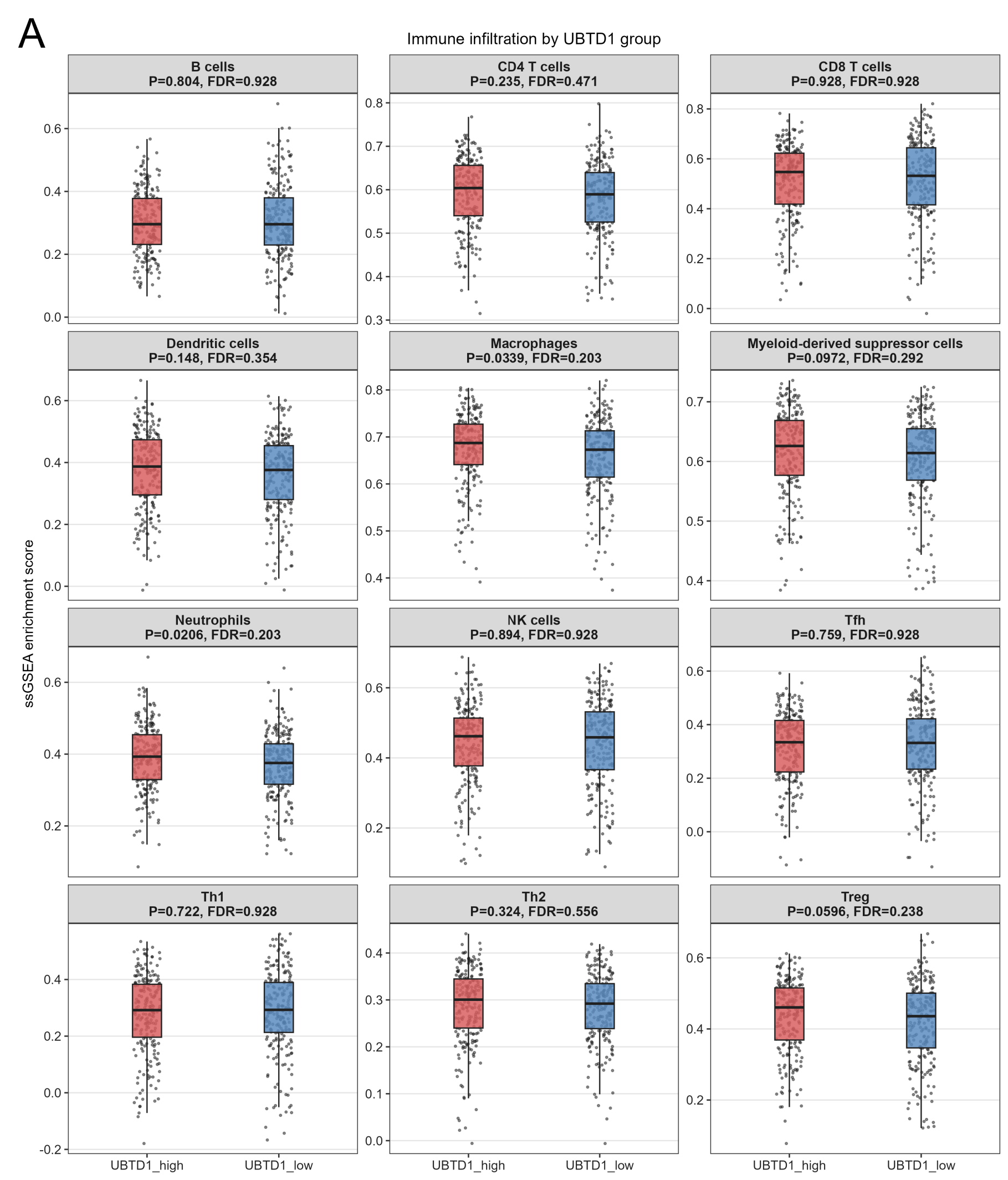


Supplementary Figure S3. Immune cell infiltration analysis stratified by UBTD1 expression in TCGA-OV cohort. Samples were stratified into UBTD1-high (n = 217) and UBTD1-low (n = 217) groups based on median UBTD1 expression. Immune cell infiltration scores were estimated using single-sample gene set enrichment analysis (ssGSEA). Comparisons of B cells, CD4 T cells, CD8 T cells, dendritic cells, macrophages, myeloid-derived suppressor cells (MDSCs), neutrophils, NK cells, T follicular helper (Tfh) cells, Th1 cells, Th2 cells, and regulatory T (Treg) cells between groups are shown. Macrophage and neutrophil infiltration were slightly elevated in the UBTD1-high group, with no notable changes in other immune populations. .Group differences were evaluated by the Wilcoxon rank-sum test, and the Benjamini-Hochberg method was applied for false discovery rate (*FDR*) correction.
