# Supplementary material for: UBTD1 Drives Ovarian Cancer Progression via Mutation‐Associated Alterations, Stromal Microenvironment Remodeling, and TNF/AP‐1 Signaling
Source: Hum Mutat. 2026 Jul 22;2026:9479429. doi: 10.1155/humu/9479429 (PMC13390016; doi:10.1155/humu/9479429)
Supplement: Supplementary file 2 — Supporting Information 2 The supporting table file contains the antibodies used in the experiments and the primer sequences used for qRT‐PCR. [file HUMU-2026-9479429-s002.docx]

**Supplementary Table**

Table 1 Antibodies used in the experiments.

| Antibody Name | Supplier | Catalog number | Dilution |
| --- | --- | --- | --- |
| UBTD1 Polyclonal antibody | Proteintech | 20158-1-AP | 1:1000 |
| Anti-BrdU Antibody | abcam | AB6326 | 1:500 |
| Anti-β-Actin Antibody | Merck | A5441-2ml | 1:5000 |
| Goat anti-Rat Secondary Antibody | Thermo | A11007 | 1:1000 |
| p21 Rabbit Monoclonal Antibody | CST | 2947S | 1:1000 |
| Cyclin D1 Rabbit Monoclonal Antibody | CST | 55506S | 1:1000 |
| Anti-E Cadherin Antibody | abcam | AB23130 | 1:1000 |
| Anti-N Cadherin Antibody | abcam | AB18203 | 1:1000 |
| Anti-SNAI1 Rabbit mAb | Yamei Biology | R013727 | 1:1000 |
| c-Fos Rabbit Monoclonal Antibody | CST | 2250S | 1:1000 |
| Goat anti-Mouse IgG (H+L) Secondary Antibody | Thermo | 31430 | 1:1000 |
| Goat anti-Rabbit IgG (H+L) Secondary Antibody | Thermo | 31460 | 1:1000 |

Table 2 Primer sequences used in the experiments.

| Name | Type | Primer sequences（5’-3’） |
| --- | --- | --- |
| β-actin | Forward | ATCCACGAAACTACCTTCAA |
|  | Reverse | ATCCACACGGAGTACTTGC |
| UBTD1 | Forward | AGAGCGACTACCCCATGACTG |
|  | Reverse | GGCATCCCAGATCTCCTTGC |
| TNF | Forward | CAAGGACAGCAGAGGACCAG |
|  | Reverse | TCCTTTCCAGGGGAGAGAGG |
| TNFR1 | Forward | TGCTGTACCAAGTGCCACAA |
|  | Reverse | CTGAGGCAGTGTCTGAGGTG |
| TRADD | Forward | GGAAGCGGCGGAGTAGAG |
|  | Reverse | TCTCACCTCCTGCTGCACTA |
| FOS | Forward | AGACCGAGATTGCCAACCTG |
|  | Reverse | CATCAGGGATCTTGCAGGCA |
| FOSB | Forward | GAGCTGACCGACCGACTCC |
|  | Reverse | CGGCAAATCTCTCACCTCCG |
| JUN | Forward | GTGCCGAAAAAGGAAGCTGG |
|  | Reverse | CTGCGTTAGCATGAGTTGGC |
